# Supplementary material for: Electron-Induced Decomposition of 5-Bromo-4-thiouracil and 5-Bromo-4-thio-2′-deoxyuridine: The Effect of the Deoxyribose Moiety on Dissociative Electron Attachment
Source: Int J Mol Sci. 2023 May 13;24(10):8706. doi: 10.3390/ijms24108706 (PMC10217871; doi:10.3390/ijms24108706)
Supplement: Supplementary file 1 [file ijms-24-08706-s001.zip › Supplementary Materials_proof_correctedas_md-SD.pdf]

Supplementary Information

Electron-Induced Decomposition of 5-Bromo-4-thiouracil and 5-Bromo-4-thio-2`-deoxyuridine: The Effect of the Deoxyribose Moiety on Dissociative Electron Attachment

Farhad Izadi [1,2], Adrian Szczyrba [3], Magdalena Datta[3], Olga Ciupak[4], Sebastian Demkowicz[4], Janusz Rak[3,*] and Stephan Denifl [1,2,*]

[1] Universität Innsbruck, Institut für Ionenphysik und Angewandte Physik, Technikerstrasse 25, A-6020 Innsbruck, Austria
[2] Universität Innsbruck, Center for Molecular Biosciences Innsbruck, Technikerstrasse 25, A-6020 Innsbruck, Austria.
[3] Laboratory of Biological Sensitizers, Department of Physical Chemistry, Faculty of Chemistry, University of Gdańsk, Wita Stwosza 63, 80-308 Gdańsk, Poland; adrian.szczyrba@phdstud.ug.edu.pl
[4] Department of Organic Chemistry, Faculty of Chemistry, Gdańsk University of Technology, Narutowicza 11/12, 80-233 Gdańsk, Poland
[*] Correspondence: Stephan: stephan.denifl@uibk.ac.at; Janusz: janusz.rak@ug.edu.pl

5-bromo-4-thiouracil was prepared from 5-bromouracil according to a published procedure described by Łapucha [S1] in 74% yield (lit. yield 95%). ^{1}H NMR (500 MHz, DMSO- d_6): 12.87 (s, 1H, NH), 11.93 (s, 1H, NH), 8.05 (s, 1H, CH); ^{13}C NMR (500 MHz, DMSO- d_6): 188.10, 148.68, 140.56, 106.32.

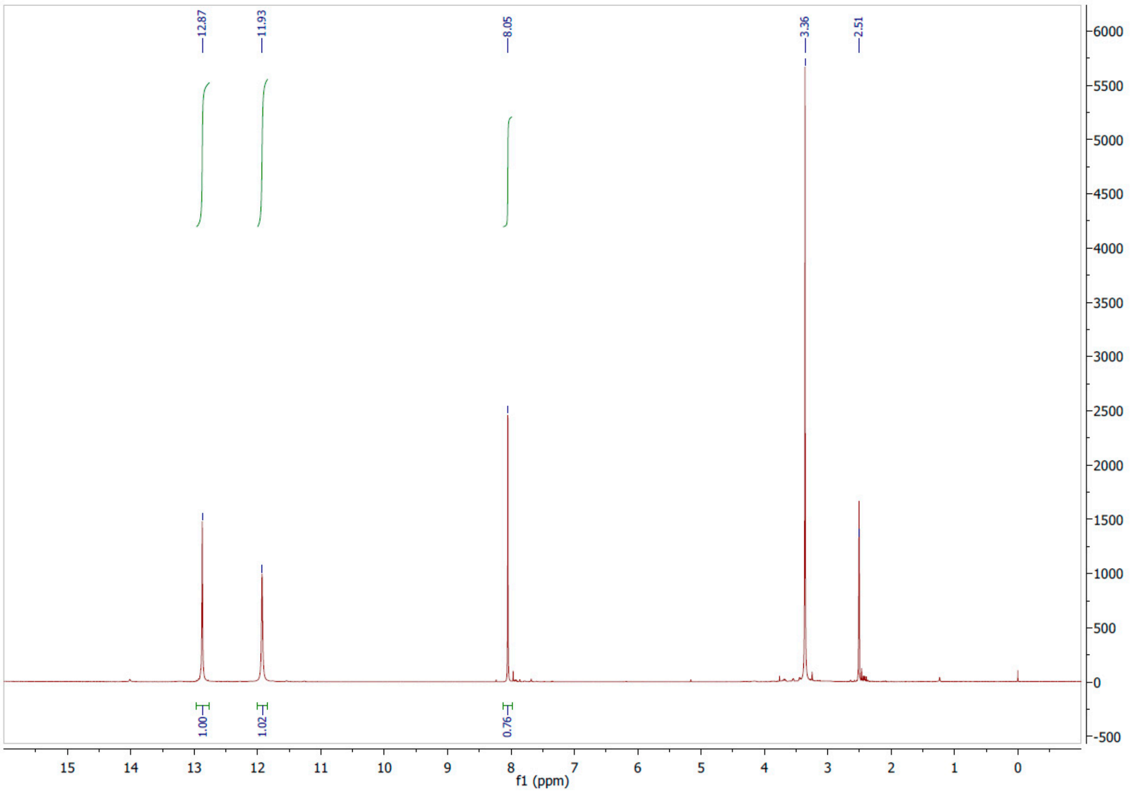

Figure S1 *^{1}H NMR spectrum of BrSU.*

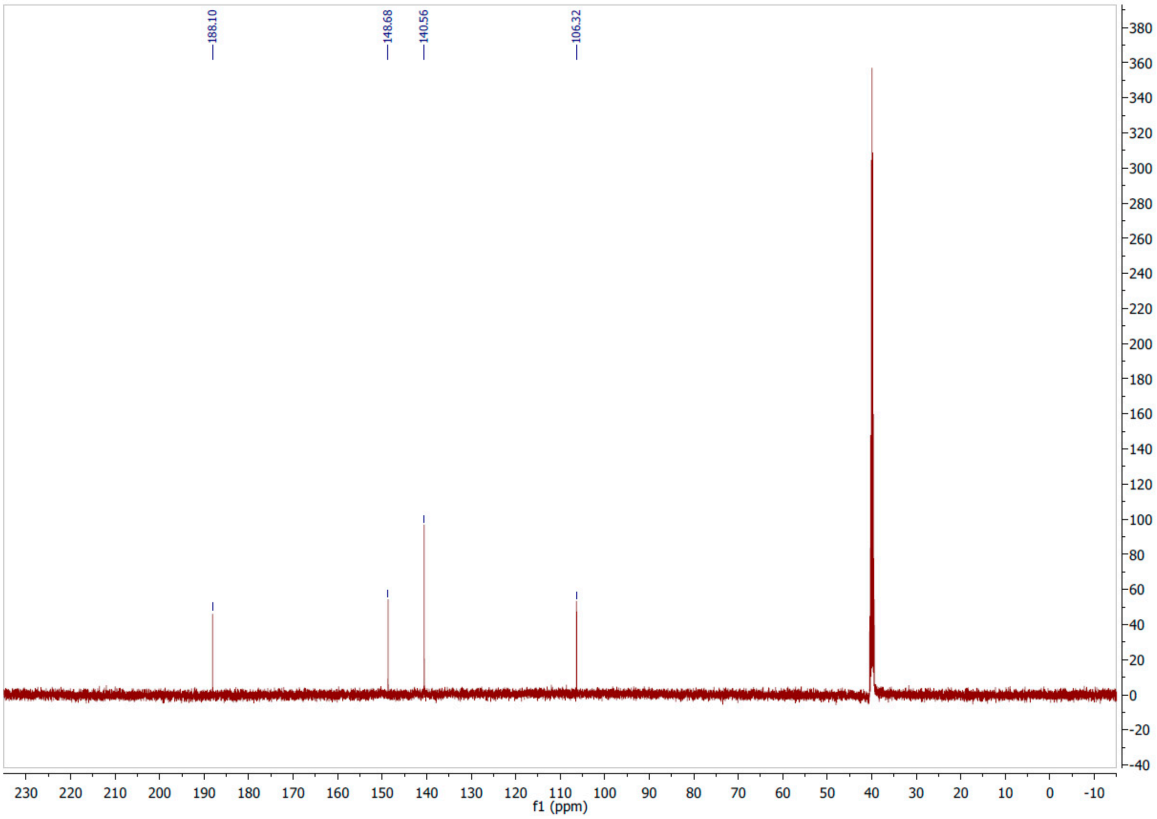

Figure S2 *^{13}C NMR spectrum of BrSU.*

5-bromo-4-thio-2’-deoxyuridine was prepared from 5-bromo-2’-deoxyuridine according to a published procedure described by Spisz et al. [S2] in 46% (lit. yield 41%).

^{1}H NMR (500 MHz, DMSO- d_6): 13.10 (s, 1H, NH), 8.54 (s, 1H, CH), 6.03 (t, *J* = 6.1 Hz, 1H, CH), 5.30 (d, *J* = 3.7 Hz, 1H, OH), 5.25 (t, *J* = 4.5 Hz, 1H, OH), 4.27 – 4.22 (m, 1H, CH), 3.82 (q, *J* = 3.2 Hz, 1H, CH), 3.62 (ddt, *J* = 15.2, 11.9, 3.4 Hz, 2H, CH_2), 2.16- 2.25 (m, 2H, CH_2); ^{13}C NMR (500 MHz, DMSO): 186.82, 147.64, 137.75, 107.09, 88.23, 86.08, 69.86, 60.82, 40.81.

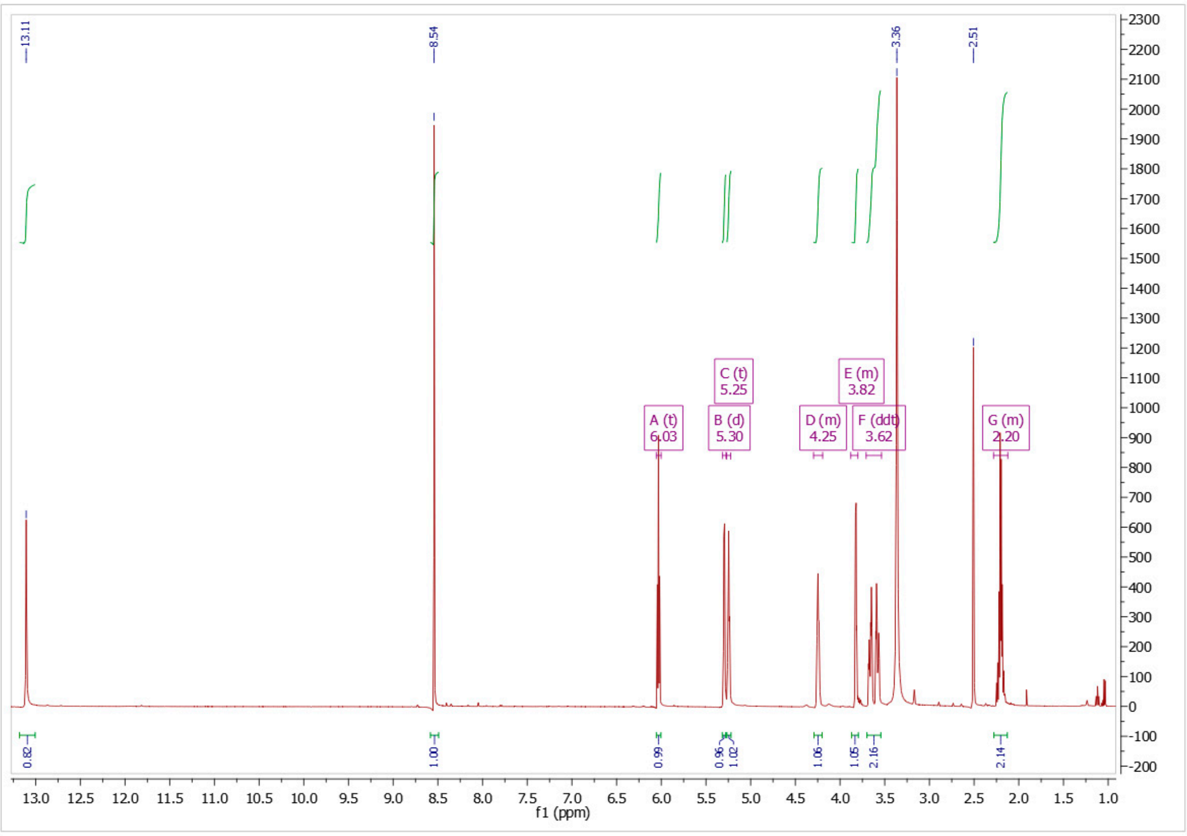

Figure S3 *^{1}H NMR spectrum of BrSdU.*

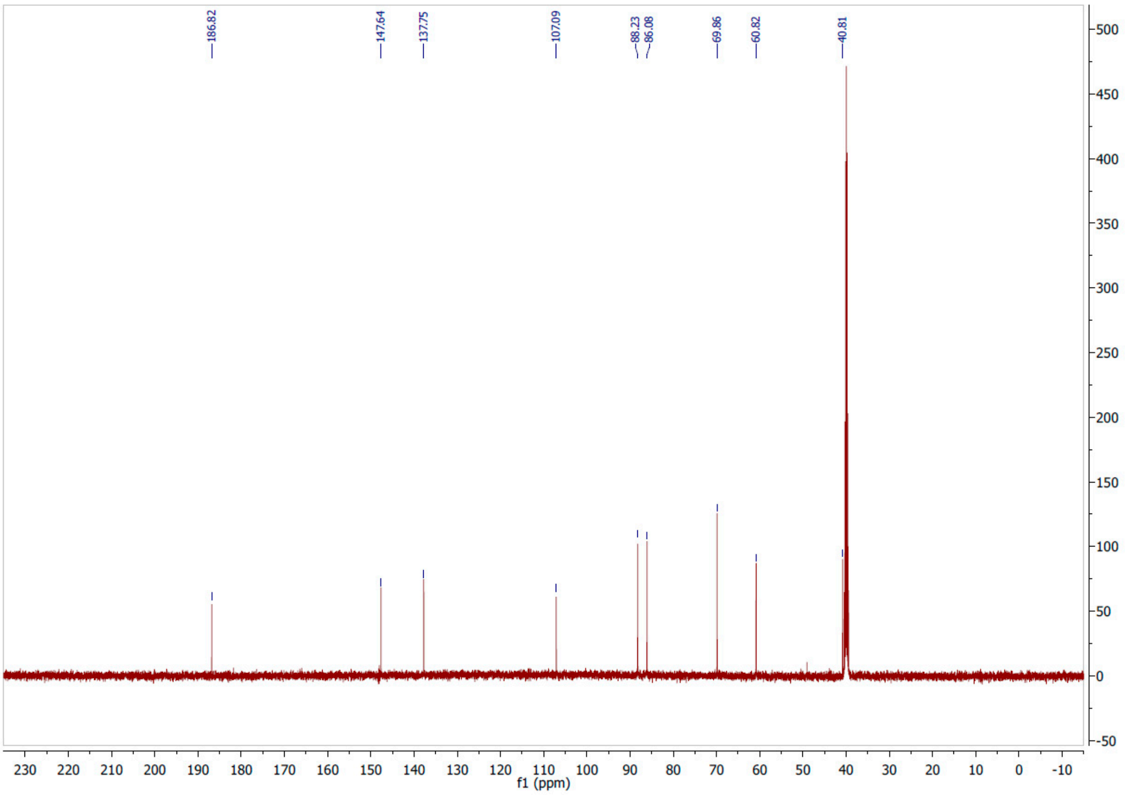

Figure S4 *^{13}C NMR spectrum of BrSdU.*

Table S1. Peak positions, experimental thresholds for the formations of fragment anions resulting from DEA to BrSdU.

Mass (m/z)	Anion	Peak Positions (eV)					Threshold (eV)
		1	2	3	4	5	Exp.
**205**	(BrSdU-deoxyribose)$^-$	≈ 0	0.18	0.5	1.0	--	≈ 0
**190**	(BrSdU- deoxyribose-NH)$^-$	≈ 0	--	--	--	--	≈ 0
**127**	(BrSdU- deoxyribose-Br+H)$^-$	≈ 0	0.16	0.54	--	--	≈ 0
**126**	(BrSdU- deoxyribose-Br)$^-$	≈ 0	0.16	0.7	--	--	≈ 0
**98**	$(C_5H_6O_2)^-$	≈ 0	0.9	2.5	4.5	5.4	≈ 0
**79**	Br^-	≈ 0	0.3	0.8	1.0	5.0	≈ 0
**42**	NCO^-	≈ 0	3.2	5.2	--	--	≈ 0
**33**	SH^-	≈ 0	0.3	1.5	2.4	5.1	≈ 0

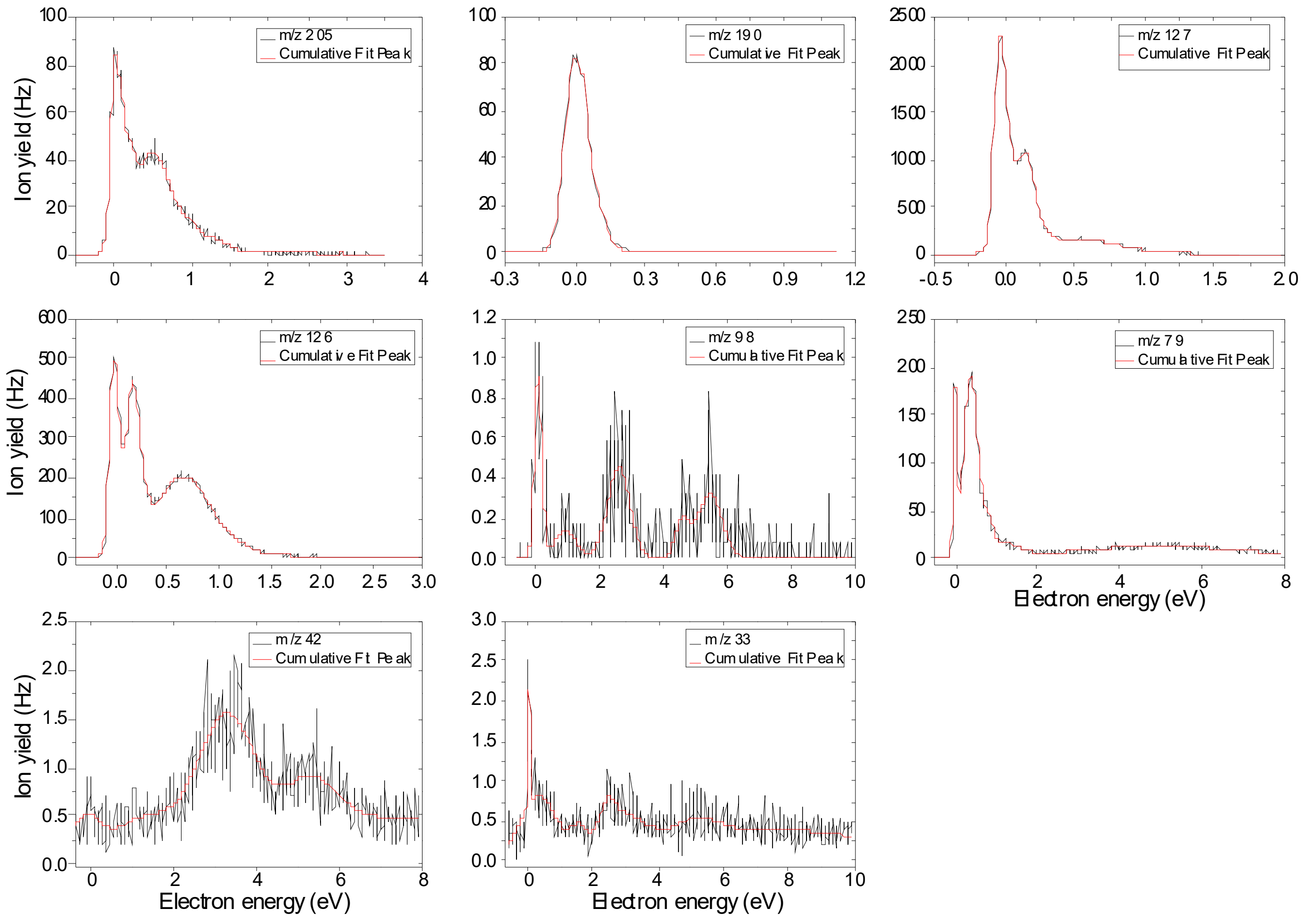

Figure S5. Anion efficiency curves of anions observed from electron attachment to molecules sublimed in a thermally heated 5-bromo-4-thio-2`-deoxyuridine (BrSdU) sample.

Cartesian coordinates of BrSU and its degradation products as well as transition states for proton transfer reactions optimized at the M062X/aug-cc-pvtz level (in Å) with the sum of electronic and thermal enthalpies energies (in Hartree) and charge and multiplicity given in square brackets. Numbers in the round brackets correspond to reaction numbers in Figure 2.

BrSU
[0 1]

N	-0.003972	0.016000	0.011155
C	0.011494	-0.002853	1.394278
C	1.353820	0.003137	1.947766
C	2.420655	0.024857	1.130182
N	2.284577	0.042131	-0.229001
C	1.059401	0.038941	-0.866543
S	-1.388713	-0.027673	2.243785
Br	1.613325	-0.019502	3.802256
O	0.937605	0.053891	-2.065100
H	3.432161	0.029706	1.507897
H	-0.916164	0.013016	-0.425510
H	3.089524	0.058291	-0.834108

SUM OF ELECTRONIC AND THERMAL ENTHALPIES (Ha) = -3311.32464

BrSU (1)
[-1 2]

N	0.004466	0.016127	0.002576
C	0.039395	-0.002860	1.431488
C	1.329342	0.002837	1.942552
C	2.463777	0.024991	1.148542
N	2.284263	0.042293	-0.248961
C	1.060027	0.038021	-0.858682
S	-1.464226	-0.028184	2.228632
Br	1.611476	-0.019671	3.813882
O	0.937789	0.054439	-2.078640
H	3.471513	0.030040	1.521031
H	-0.912869	0.013026	-0.411489
H	3.068761	0.058881	-0.873877

SUM OF ELECTRONIC AND THERMAL ENTHALPIES (Ha) = -3311.368042

BrSU-H (2a)
[-1 1]

N	-0.003511	0.000182	-0.029555
C	0.040962	0.000383	1.382324
N	1.259080	0.000137	1.960618
C	2.409409	-0.000400	1.272748
C	2.323244	-0.000259	-0.192732
C	1.120340	-0.000109	-0.788520
O	-1.037244	0.000686	1.967826
S	3.908467	0.000193	2.046599
Br	3.850144	-0.000286	-1.312476

H	-0.920469	0.000339	-0.441496
H	0.996481	-0.000109	-1.861974

SUM OF ELECTRONIC AND THERMAL ENTHALPIES (Ha) = -3310.859677

BrSU-H (2b)
[-1 1]

C	-0.005026	0.002028	0.022239
N	0.007071	0.014073	1.392803
C	1.121955	-0.003117	2.226612
C	2.346052	-0.037540	1.514765
C	2.477000	-0.053232	0.134564
N	1.262140	-0.031877	-0.518074
S	0.832053	0.018754	3.888542
Br	3.951284	-0.064346	2.541723
O	-1.038401	0.019426	-0.642608
H	-0.895061	0.039201	1.850594
H	1.260842	-0.041029	-1.529215

SUM OF ELECTRONIC AND THERMAL ENTHALPIES (Ha) = -3310.773086

H (2a and 2b)
[0 2]

H	0.000000	0.000000	0.000000

SUM OF ELECTRONIC AND THERMAL ENTHALPIES (Ha) = -0.495126

BrSU-Br (3a)
[-1 1]

N	-0.079598	-0.150695	-0.051873
C	-0.014383	0.025055	1.298914
N	1.267912	0.093072	1.869292
C	2.466855	0.003825	1.222816
C	2.345515	-0.175827	-0.162797
C	1.071740	-0.240731	-0.701844
O	-0.981970	0.127042	2.044321
S	3.913684	0.111895	2.104298
H	0.979008	-0.379927	-1.777997
H	1.292219	0.223332	2.869832
H	3.230766	-0.259053	-0.770770

SUM OF ELECTRONIC AND THERMAL ENTHALPIES (Ha) = -737.167098

BrSU-Br (3b)
[-1 1]

C	-0.023971	-0.004212	-0.001297
N	-0.002879	-0.091976	1.367432
C	1.123414	-0.010889	2.189542
C	2.432257	-0.131214	1.593649
C	2.387251	-0.097480	0.246606
N	1.220697	0.043775	-0.538913
S	0.799326	0.211598	3.824099
O	-1.062071	0.031139	-0.655915
H	3.267101	-0.194482	-0.386475

H	-0.908209	-0.072157	1.815140
H	1.253795	0.102208	-1.543185

SUM OF ELECTRONIC AND THERMAL ENTHALPIES (Ha) = -737.123747

BrSU-Br (3c)
[-1 1]

N	0.038264	-0.125330	-0.019401
C	0.056733	0.060208	1.379959
N	1.260525	0.211489	1.977281
C	2.400609	0.188724	1.278759
C	2.366141	-0.003291	-0.166087
C	1.176413	-0.154556	-0.765974
O	-1.032504	0.069440	1.947783
S	3.921442	0.377783	2.015849
H	1.054807	-0.302271	-1.831418
H	-0.873159	-0.234994	-0.427405
H	3.289122	-0.020361	-0.720951

SUM OF ELECTRONIC AND THERMAL ENTHALPIES (Ha) = -737.086097

Br (3a, 3b and 3c)
[0 2]

Br	0.000000	0.000000	0.000000

SUM OF ELECTRONIC AND THERMAL ENTHALPIES (Ha) = -2574.19555

BrSU-HBr (4a)
[-1 2]

C	0.018117	-0.000456	0.010045
N	-0.004588	0.000842	1.382999
C	1.099525	-0.000378	2.235057
C	2.304066	-0.003304	1.533661
C	2.463235	-0.004798	0.211974
N	1.286728	-0.003275	-0.518333
S	0.810142	0.001549	3.897010
O	-1.001456	0.000795	-0.669520
H	-0.918843	0.002905	1.810986
H	1.301310	-0.004218	-1.525992

SUM OF ELECTRONIC AND THERMAL ENTHALPIES (Ha) = -736.487877

BrSU-HBr (4b)
[-1 2]

C	0.018117	-0.000456	0.010045
N	-0.004588	0.000842	1.382999
C	1.099525	-0.000378	2.235057
C	2.304066	-0.003304	1.533661
C	2.463235	-0.004798	0.211974
N	1.286728	-0.003275	-0.518333
S	0.810142	0.001549	3.897010
O	-1.001456	0.000795	-0.669520
H	-0.918843	0.002905	1.810986
H	1.301310	-0.004218	-1.525992

SUM OF ELECTRONIC AND THERMAL ENTHALPIES (Ha) = -736.448703

BrSU-HBr (4c)
[-1 2]

C	-0.007131	0.000672	0.044181
N	-0.086606	0.000213	1.400451
C	1.005554	-0.000030	2.172642
C	2.284767	0.000222	1.529862
C	2.424995	0.000571	0.208802
N	1.275028	0.000733	-0.543610
S	0.973164	-0.000234	3.868829
O	-0.966771	0.000609	-0.721616
H	3.365615	0.000650	-0.326557
H	1.290810	0.000911	-1.549067

SUM OF ELECTRONIC AND THERMAL ENTHALPIES (Ha) = -736.469680

HBr (4a, 4b and 4c)
[0 1]

Br	0.000000	0.000000	-0.205989
H	0.000000	0.000000	1.213989

SUM OF ELECTRONIC AND THERMAL ENTHALPIES (Ha) = -2574.829103

Br (5)
[-1 1]

Br	0.000000	0.000000	0.000000

SUM OF ELECTRONIC AND THERMAL ENTHALPIES (Ha) = -2574.321536

BrSU-Br (5)
[0 2]

C	-0.022598	0.003204	0.000949
N	-0.028097	0.017262	1.386494
C	1.063841	-0.007436	2.235870
C	2.292399	-0.052349	1.525136
C	2.396207	-0.068928	0.201589
N	1.242777	-0.041299	-0.554524
S	0.919722	0.013562	3.870729
O	-1.029397	0.027542	-0.661488
H	3.328696	-0.103016	-0.343445
H	-0.946448	0.049791	1.810846
H	1.269611	-0.052024	-1.561472

SUM OF ELECTRONIC AND THERMAL ENTHALPIES (Ha)= -736.998588

CNO (6a and 6b)
[-1 1]

N	-0.251725	-0.000000	0.140936
C	0.431906	-0.000000	1.104182
O	1.139752	-0.000000	2.101548

SUM OF ELECTRONIC AND THERMAL ENTHALPIES (Ha)= -168.121922

BrSU-CN1O (6a)

[0 2]
H 0.108382 -0.017127 -0.050569
N -0.066112 -0.009490 0.952427
S 2.605496 0.011339 0.943085
C 0.992906 0.005075 1.642546
Br 2.561183 0.039294 4.052849
C 0.902042 0.017504 3.131467
H -0.289560 0.022989 4.862897
C -0.245492 0.013812 3.784390
H -1.161905 0.001170 3.209158

SUM OF ELECTRONIC AND THERMAL ENTHALPIES (Ha) = -3143.15486

BrSU-CN3O (6b)
[0 2]
S 2.278988 0.882916 0.017143
C 0.670867 1.297364 -0.004330
C -0.408905 0.433171 -0.069249
Br -0.208237 -1.419172 -0.145580
H -2.560361 0.137858 -0.136649
C -1.795692 0.913055 -0.082700
H -3.050297 2.325518 -0.051443
N -2.046833 2.156366 -0.033596
H 0.379736 2.342775 0.034655

SUM OF ELECTRONIC AND THERMAL ENTHALPIES (Ha) = -3143.161147

SH (7)
[-1 1]
S 0.000000 0.000000 -0.167885
H 0.000000 0.000000 1.175885

SUM OF ELECTRONIC AND THERMAL ENTHALPIES (Ha) = -398.813215

BrSU-SH (7)
[0 2]
C -0.013412 -0.000288 0.045135
N -0.008039 0.000861 1.444897
C 1.070345 -0.000628 2.077287
C 2.383557 -0.003516 1.557947
C 2.431966 -0.004746 0.204312
N 1.281261 -0.003186 -0.508278
Br 3.919398 -0.005480 2.633070
O -0.983294 0.000939 -0.658934
H 3.356663 -0.006948 -0.357139
H 1.308323 -0.004078 -1.517082

SUM OF ELECTRONIC AND THERMAL ENTHALPIES (Ha) = -2912.447725

TS (N1 to O2)
[-1 2]
C 2.469324 -0.246633 -0.000047
N 1.767625 0.885006 -0.000213

C	0.321181	0.881010	-0.000067
C	-0.214753	-0.399304	-0.000027
C	0.532991	-1.579255	-0.000069
N	1.925884	-1.445227	0.000001
S	-0.414222	2.416260	0.000060
Br	-2.099507	-0.603923	0.000013
O	3.756967	-0.394993	0.000150
H	0.101166	-2.563220	0.000004
H	2.215894	1.785697	-0.000145
H	3.230470	-1.598762	0.000252

SUM OF ELECTRONIC AND THERMAL ENTHALPIES (Ha) = -3311.301792

TS (N3 to S4)
[-1 2]

C	-0.015628	0.000590	0.090105
N	-0.007851	0.000340	1.440450
C	1.182876	0.000181	2.173855
C	2.400494	0.000282	1.552555
C	2.466503	0.000544	0.163224
N	1.246579	0.000719	-0.520060
S	0.643061	-0.000159	3.835889
Br	4.017589	0.000064	2.540791
O	-1.014332	0.000716	-0.624786
H	3.377319	0.000637	-0.408442
H	-0.530341	0.000062	2.759070
H	1.219762	0.000850	-1.523700

SUM OF ELECTRONIC AND THERMAL ENTHALPIES (Ha) = -3311.316193

TS (S4 to C5)
[-1 2]

C	-0.120079	-0.432617	-0.085141
N	-0.314464	-0.453297	1.280284
C	0.679188	-0.054833	2.020910
C	1.953179	0.403039	1.575838
C	2.162622	0.430621	0.253731
N	1.126815	0.015363	-0.548005
S	0.809405	0.058244	3.756580
Br	4.463736	1.368183	2.350436
O	-0.954265	-0.775275	-0.906187
H	3.074635	0.751712	-0.227555
H	1.221145	0.011081	-1.550054
H	2.193777	0.545709	3.001455

SUM OF ELECTRONIC AND THERMAL ENTHALPIES (Ha) = -3311.307996

Cartesian coordinates of BrSdU and its degradation products optimized at the M062X/aug-cc-pvtz level (in Å) with thermal enthalpies H energies (in Hartree) and with charge and multiplicity (in brackets). Numbers in the round brackets correspond to reaction numbers in Figure 5.

BrSdU
[0 1]

O	-0.000883	-0.011686	0.005568
C	0.006163	-0.020314	1.399512
C	1.475524	-0.017299	1.854437
C	2.243739	0.259921	0.567122
C	1.303106	-0.304419	-0.495300
N	-0.722747	-1.226987	1.884266
C	-1.237414	-2.162706	1.036096
C	-1.868350	-3.268613	1.481625
C	-2.055231	-3.491419	2.895414
N	-1.535773	-2.465702	3.663290
C	-0.871190	-1.331851	3.252486
O	-0.444170	-0.499547	4.021135
S	-2.790220	-4.776612	3.605920
Br	-2.502006	-4.517773	0.231959
O	2.382086	1.661519	0.457533
C	1.505088	-1.792741	-0.717591
O	0.532199	-2.225486	-1.643632
H	-0.545865	0.841399	1.767713
H	3.219523	-0.233013	0.554295
H	1.387382	0.214699	-1.450707
H	2.778996	1.885050	-0.388088
H	2.518448	-1.954412	-1.098821
H	1.411868	-2.329842	0.234545
H	0.575075	-3.179856	-1.741708
H	-1.644385	-2.555517	4.664801
H	-1.094793	-1.963609	-0.016659
H	1.746255	-0.982371	2.278449
H	1.666841	0.748060	2.599135

SUM OF ELECTRONIC AND THERMAL ENTHALPIES (Ha) = -3732.187213

BrSdU-deoxyribiose (1)
[-1 1]

Br	0.006036	0.002788	-0.013111
S	-0.015229	0.010917	3.435823
C	1.523838	0.008024	1.113438
H	2.853220	0.006585	-0.541329
C	2.788828	0.009019	0.543918
C	1.396628	0.010986	2.513142
H	2.599891	0.017043	4.148677
N	2.616163	0.014780	3.139488
O	4.867172	0.018529	3.291742
C	3.890005	0.015252	2.555980

N 3.938569 0.012316 1.189861

SUM OF ELECTRONIC AND THERMAL ENTHALPIES (Ha) = -3310.821721

Deoxyribose (1)
[0 2]
H -3.438788 -0.100088 -0.381515
O -2.712025 0.178828 0.177747
H -1.591518 1.289307 -1.165644
C -1.549238 0.349375 -0.604115
H -1.418248 -0.475735 -1.312339
H -0.562425 1.081180 1.146085
C -0.356138 0.385235 0.331127
H 0.798070 1.399619 -1.226292
H 1.612442 1.984943 0.929655
O 1.940269 1.196997 0.489183
O -0.134850 -0.894433 0.916310
C 0.952501 0.705222 -0.396623
C 1.411011 -0.678812 -0.861457
H 2.498596 -0.741242 -0.829400
H 1.096498 -0.863287 -1.892909
H 1.145410 -2.471761 0.565158
C 0.727332 -1.586698 0.114613

SUM OF ELECTRONIC AND THERMAL ENTHALPIES (Ha) = -421.378387

BrSdU-deoxyribiose-NH (2)
[-1 1]
O -0.646945 0.013752 0.636187
C -0.158005 0.003269 1.730639
N 1.229857 -0.032876 2.000288
C 1.299941 -0.031904 3.306101
H 2.262328 -0.056282 3.808913
C 0.062847 0.002162 4.005921
Br -0.087878 0.009819 5.879592
S -2.609857 0.070202 3.202829
C -0.957160 0.027143 3.082387

SUM OF ELECTRONIC AND THERMAL ENTHALPIES (Ha) = -3255.426079

Deoxyribose-NH (2)
[0 2]
H -3.386329 -0.591191 0.389498
O -2.617885 -0.667336 -0.179163
H -1.457778 -1.682417 1.212226
C -1.454340 -0.764369 0.613893
H -1.371047 0.089728 1.297343
H -0.363625 -1.622135 -1.015314
C -0.251401 -0.792764 -0.313678
H 0.975592 -1.478124 1.365387
H 1.919166 -2.145984 -0.732569
O 2.137194 -1.297086 -0.339987
O -0.141130 0.391257 -1.079949

C	1.061430	-0.858718	0.468048
C	1.307705	0.607277	0.797706
H	2.372242	0.822679	0.800244
H	0.893360	0.875740	1.766892
H	1.255589	1.867756	-1.002152
C	0.555842	1.370112	-0.319651
N	-0.286137	2.393231	0.217213
H	-1.175056	2.327420	-0.287155

SUM OF ELECTRONIC AND THERMAL ENTHALPIES (Ha) = -476.710084

BrSdU-deoxyribose-Br+H (3a)
[-1 1]

N	-0.005727	-0.314545	-0.081771
C	0.147771	-0.356879	1.224702
C	1.309418	-0.253976	2.053367
C	2.379968	-0.082763	1.233839
N	2.299755	-0.025632	-0.146046
C	1.115587	-0.140188	-0.848503
S	-1.368824	-0.585305	2.152750
O	1.122633	-0.082402	-2.078334
H	3.403194	0.026154	1.590270
H	-2.185271	-0.632461	1.088254
H	3.119918	0.102167	-0.717131

SUM OF ELECTRONIC AND THERMAL ENTHALPIES (Ha) = -737.13121

BrSdU-deoxyribose-Br+H (3b)
[-1 1]

N	0.043881	-0.218195	-0.068840
C	0.155090	-0.373673	1.291590
C	1.311676	-0.276774	1.990396
C	2.361251	0.015452	1.066787
N	2.331561	0.204796	-0.244486
C	1.130982	0.088540	-0.883097
S	-1.366648	-0.760618	2.157384
O	0.944492	0.225709	-2.091701
H	3.371603	0.114291	1.469803
H	-0.829268	-0.328334	-0.560833
H	-2.239463	-0.529771	1.160913

SUM OF ELECTRONIC AND THERMAL ENTHALPIES (Ha) = -737.123747

BrSdU-deoxyribose-Br+H (3c)
[-1 1]

N	0.023561	-0.062536	-0.074981
C	0.074202	-0.298418	1.300822
C	1.337117	-0.163051	1.985962
C	2.370950	-0.022381	1.132071
N	2.283031	-0.008690	-0.278524
C	1.093084	0.026307	-0.929241
S	-1.393747	-0.711564	2.008431
O	0.973347	0.121786	-2.147405

H	3.401498	0.114387	1.453970
H	-0.882549	-0.098214	-0.520148
H	3.097171	0.059847	-0.866373

SUM OF ELECTRONIC AND THERMAL ENTHALPIES (Ha) = -737.086097

Deoxyribose-Br+H (3a, 3b and 3c)
[0 2]

C	0.061617	0.018902	-0.044090
O	0.026093	0.228437	1.292761
C	1.347315	0.051392	1.843931
C	2.085447	-0.819335	0.827764
C	1.432559	-0.406281	-0.489639
C	1.967777	1.424896	2.025503
O	1.173494	2.242142	2.846963
O	1.832444	-2.196334	1.011571
Br	-0.889307	1.308036	-1.071557
H	0.286382	2.263890	2.472606
H	2.938747	1.325705	2.511685
H	2.124471	1.885301	1.041612
H	1.220386	-0.439768	2.808849
H	3.159072	-0.612726	0.835629
H	2.154465	-2.473415	1.873022
H	1.403283	-1.239387	-1.188956
H	1.970193	0.420660	-0.958471

SUM OF ELECTRONIC AND THERMAL ENTHALPIES (Ha) = -2995.039554

BrSdU-deoxyribose-Br (4a)
[-1 2]

C	0.055891	-0.009014	-0.028791
H	0.000194	0.021932	2.105362
C	0.625676	0.004496	1.216016
N	1.940349	-0.001812	1.438361
O	3.988842	-0.031384	0.398436
C	2.763448	-0.023487	0.350091
H	2.786590	-0.055301	-1.707497
N	2.155243	-0.038479	-0.919039
S	0.239801	-0.052801	-2.785227
C	0.811715	-0.032813	-1.188969

SUM OF ELECTRONIC AND THERMAL ENTHALPIES (Ha) = -736.487965

BrSdU-deoxyribose-Br (4b)
[-1 2]

C	0.020854	-0.018380	-0.055824
N	0.126424	-0.563576	1.232360
C	1.306603	-0.638113	1.789270
C	2.451616	-0.200660	1.158254
C	2.324889	0.324317	-0.109398
N	1.162410	0.426186	-0.722754
S	1.427388	-1.336226	3.434591
O	-1.083016	0.062638	-0.589561

H 3.197312 0.678956 -0.655793
H 0.113035 -1.583953 3.512336

SUM OF ELECTRONIC AND THERMAL ENTHALPIES (Ha) = -736.454184Deoxyribose-Br (4a and 4b)
[0 1]
H -0.539824 2.255809 0.589319
O 0.161745 2.711765 0.110823
H 1.657690 2.277005 -1.168631
C 0.862265 1.753411 -0.637256
H 0.212480 1.282662 -1.383968
H 2.256787 1.132832 0.887925
C 1.486510 0.689172 0.257029
H 2.370206 -0.236777 -1.507761
H 3.702783 -0.738152 0.381925
O 2.932479 -1.280392 0.194174
O 0.513651 0.142078 1.154667
C 1.981120 -0.522106 -0.526619
C 0.714203 -1.370612 -0.641052
H 0.962904 -2.425733 -0.557748
H 0.206411 -1.203590 -1.585895
H -0.383688 -1.659193 1.278611
C -0.139399 -0.902784 0.544079
Br -1.963912 -0.318713 -0.073948

SUM OF ELECTRONIC AND THERMAL ENTHALPIES (Ha) = -2995.687726

$C_5H_6O_2$ (5)
[-1 2]
H 0.968002 -1.331174 0.168854
O 0.101347 -1.463156 0.665738
H 1.141984 0.743400 1.729169
H -1.096972 -0.086433 1.579776
C -0.464949 -0.169379 0.685695
C 0.655134 0.886399 0.743564
H 0.223048 1.890902 0.726752
H 2.192790 1.590436 -0.716311
C 1.634012 0.703261 -0.389042
O 2.138843 -0.484533 -0.574390
C -1.341346 0.028878 -0.522533
H -0.839794 -0.132024 -1.471476
C -2.621366 0.373345 -0.477744
H -3.211256 0.511999 -1.375771
H -3.123299 0.532792 0.471222

SUM OF ELECTRONIC AND THERMAL ENTHALPIES (Ha) = -345.600668

BrSdU- $C_5H_6O_2$ (5)
[0 1]
Br -0.263004 0.166273 0.153892
S 0.109953 -0.006901 3.516651
C 1.370708 0.076587 1.062308
H 2.563943 0.129416 -0.725166

C 2.519706 0.078634 0.352480
C 1.401526 0.005559 2.506637
H 2.766997 -0.106882 4.028533
N 2.690994 -0.055741 3.020858
O 4.984364 -0.111412 2.838939
C 3.878969 -0.056182 2.342171
N 3.716978 0.014270 0.980198
O 4.860436 0.019443 0.222616
H 5.561518 -0.034326 0.900203

SUM OF ELECTRONIC AND THERMAL ENTHALPIES (Ha) = -3386.487056

Br (6)
[-1 1]
Br 0.000000 0.000000 0.000000

SUM OF ELECTRONIC AND THERMAL ENTHALPIES (Ha) = -2574.321536

BrSdU-Br (6)
[0 2]
O -0.007645 -0.049058 0.012364
C 0.017669 -0.041007 1.408334
C 1.492326 -0.021711 1.843278
C 2.237884 0.272397 0.547373
C 1.295899 -0.312191 -0.502875
N -0.701172 -1.241638 1.912923
C -1.257107 -2.185961 1.075415
C -1.897707 -3.227210 1.603698
C -2.091921 -3.443868 2.987807
N -1.533701 -2.427891 3.742058
C -0.845269 -1.319185 3.284661
O -0.393939 -0.478760 4.031776
O 2.343207 1.676991 0.436032
C 1.526238 -1.795425 -0.725819
O 0.558043 -2.248948 -1.648396
H -0.531514 0.825148 1.771578
H 3.224445 -0.198114 0.521179
H 1.359094 0.207201 -1.459944
H 2.720030 1.909276 -0.416368
H 2.540659 -1.936864 -1.111918
H 1.445337 -2.334108 0.226548
H 0.687717 -3.184770 -1.818193
H -1.121617 -2.010742 0.016327
H 1.779800 -0.986489 2.256927
H 1.683720 0.741135 2.590434
H -1.633980 -2.484596 4.747627
S -2.878912 -4.719609 3.667602

SUM OF ELECTRONIC AND THERMAL ENTHALPIES (Ha) = -1157.860404

NCO (7a and 7b)
[-1 1]
N -0.251702 -0.000000 0.140918

C	0.431929	-0.000000	1.104215
O	1.139704	-0.000000	2.101535

SUM OF ELECTRONIC AND THERMAL ENTHALPIES (Ha) = -168.121953

BrSdU-deoxyribose-NCO (7a)
[0 1]

Br	-1.875990	-0.591891	0.000013
S	-0.027810	2.376987	0.000121
C	-0.030196	-0.427170	-0.000073
H	0.728199	-2.467223	0.000104
C	0.876451	-1.400851	-0.000025
C	0.651251	0.908302	-0.000294
H	2.711831	1.306256	0.000276
N	1.983878	0.609690	-0.000273
O	3.305303	-1.286278	0.000217
C	2.224995	-0.768984	-0.000042

SUM OF ELECTRONIC AND THERMAL ENTHALPIES (Ha) = -3255.959395

BrSdU-BrSU-NCO (7b)
[0 2]

H	-2.687362	-1.307623	0.240290
H	-1.105854	-2.087110	0.048587
C	-1.697398	-1.207298	-0.202946
H	-1.801714	-1.147818	-1.285862
O	-1.899949	1.132396	-0.004302
H	-1.446663	1.958869	0.182758
H	-0.923644	-0.024999	1.405523
C	-1.023559	0.057888	0.314661
H	1.556990	-1.434051	0.075649
C	0.324921	0.268931	-0.276991
H	1.494082	-0.275012	1.405498
O	2.682408	0.281184	-0.211647
H	3.463443	-0.197618	0.072602
C	1.530052	-0.357687	0.311423
H	0.386950	0.635718	-1.294344

SUM OF ELECTRONIC AND THERMAL ENTHALPIES (Ha) = -308.063434

SH (8)
[-1 1]

S	0.000000	0.000000	-0.167884
H	0.000000	0.000000	1.175884

SUM OF ELECTRONIC AND THERMAL ENTHALPIES (Ha) = -398.813215

BrSdU-SH (8)
[0 2]

O	-0.001420	-0.027081	-0.002889
C	-0.003233	-0.043387	1.392730
C	1.462834	-0.041873	1.856628
C	2.237684	0.253345	0.577620
C	1.307413	-0.303779	-0.497644

N	-0.736566	-1.248775	1.875134
C	-1.245305	-2.159154	1.017964
C	-1.901485	-3.252886	1.491116
C	-2.002856	-3.303345	2.889036
N	-1.565545	-2.464955	3.712857
C	-0.887051	-1.332318	3.275382
O	-0.435280	-0.466222	3.982407
Br	-2.603176	-4.569331	0.348214
O	2.369347	1.656800	0.483508
C	1.523708	-1.787733	-0.733961
O	0.550961	-2.225354	-1.659342
H	-0.557466	0.815250	1.764501
H	3.216370	-0.234031	0.565928
H	1.395178	0.226693	-1.446546
H	2.774155	1.891014	-0.355403
H	2.536762	-1.934228	-1.121544
H	1.440514	-2.333926	0.213956
H	0.652360	-3.167010	-1.816860
H	-1.090071	-1.965983	-0.035865
H	1.733163	-1.010594	2.272716
H	1.645975	0.714888	2.611588

SUM OF ELECTRONIC AND THERMAL ENTHALPIES (Ha) = -3333.311519

[S1] Łapucha, A. R., A Rapid and Efficient Synthesis of Sulfur Analogues of Pyrimidine Bases. *Synthesis*, **1987**, 1987(03), 256–258.

[S2] Spisz, P.; Zdrowowicz, M.; Makurat, S.; Kozak, W.; Skotnicki, K.; Bobrowski, K.; Rak, J. Why Does the Type of Halogen Atom Matter for the Radiosensitizing Properties of 5-Halogen Substituted 4-Thio-20 -Deoxyuridines?, *Molecules* **2019**, *24*, 2819.
